# Histone Acetylation and Its Modifiers in the Pathogenesis of Diabetic Nephropathy

**DOI:** 10.1155/2016/4065382

**Published:** 2016-06-09

**Authors:** Xiaoxia Li, Chaoyuan Li, Guangdong Sun

**Affiliations:** Department of Nephrology, The Second Hospital of Jilin University, Changchun 130041, China

## Abstract

Diabetic nephropathy (DN) remains a leading cause of mortality worldwide despite advances in its prevention and management. A comprehensive understanding of factors contributing to DN is required to develop more effective therapeutic options. It is becoming more evident that histone acetylation (HAc), as one of the epigenetic mechanisms, is thought to be associated with the etiology of diabetic vascular complications such as diabetic retinopathy (DR), diabetic cardiomyopathy (DCM), and DN. Histone acetylases (HATs) and histone deacetylases (HDACs) are the well-known regulators of reversible acetylation in the amino-terminal domains of histone and nonhistone proteins. In DN, however, the roles of histone acetylation (HAc) and these enzymes are still controversial. Some new evidence has revealed that HATs and HDACs inhibitors are renoprotective in cellular and animal models of DN, while, on the other hand, upregulation of HAc has been implicated in the pathogenesis of DN. In this review, we focus on the recent advances on the roles of HAc and their covalent enzymes in the development and progression of DN in certain cellular processes including fibrosis, inflammation, hypertrophy, and oxidative stress and discuss how targeting these enzymes and their inhibitors can ultimately lead to the therapeutic approaches for treating DN.

## 1. Introduction

DN is one of the most serious diabetic microvascular complications and the leading cause of end-stage renal diseases (ESRD); it brings about heavy social and economic burden worldwide, particularly in the developed countries. Both type 1 and type 2 diabetic patients presented indistinguishable and variable pathological changes and clinical course; the prognosis is difficult to predict because of diverse pathogenesis. Clinically, DN is characterised by different degrees of proteinuria, albuminuria, increased serum creatinine (Scr), decreased glomerular filtration rate (GFR), and ESRD [1, 2]. Importantly, DN also increases the risks for the development of diabetic macrovascular complications including heart attacks and strokes [3, 4]. Pathologically, DN associated histological structural changes include glomerular mesangial expansion, glomerular basement membrane (GBM) thickening, glomerular sclerosis known as Kimmelstiel-Wilson lesions caused by excessive extracellular matrix (ECM) proteins accumulations, and tubulointerstitial fibrosis in the advanced stages [1, 5]. Arterial hyalinosis of the afferent and efferent arterioles is often prevalently caused by endothelial dysfunction and inflammation [2, 6, 7], which will lead to glomerular hyperfiltration.

In the development and progression of DN, resident kidney cells are affected by hyperglycemia: including mesangial cells, podocytes, endothelial cells, smooth muscle cells, inflammatory cells, myofibroblasts, and cells of tubular and collecting duct system [4]. Multiple contributors including environmental and genetic factors are associated with the pathogenesis of DN, which cause metabolic, hemodynamic, and biochemical changes in the diabetic kidneys [8]. Main pathways leading to DN include intracellular PKC activation and increased polyol pathway flux, production of reactive oxygen species (ROS) and advanced glycation end products (AGEs), and hypertension and glomerular hyperfiltration leading to shear stress and mechanical stretch [8, 9]. Increased blood glucose activates the renin-angiotensin system (RAS), TGF-*β*-Smad-MAPK pathway, JAK-STAT pathway, and G-protein signaling [7]; aberrant expression of ECM proteins and deregulated expression of cyclin kinases and their inhibitors; transcription factor such as NF-*κ*B [10], proinflammatory cytokines like TNF and IL-1 [11], and toll-like receptors 4 (TLR4) [12], which are considered to exert hemodynamic, proinflammatory, and profibrotic effects on kidney cells [8, 13]. There is cross-talk among the above-mentioned signaling pathways, which can amplify aberrant pathogenetic genes expression and lead to the progression of DN. In addition, the phenomenon of metabolic memory regulated by epigenetic mechanisms can promote these genes expressions [14, 15]. Although a lot of biochemical and molecular mechanisms and pathways have been broadly studied in the pathogenesis of DN, the undeniable fact is that the progressive incidence and prevalence of DN worldwide still exist, suggesting that more investigations will be needed in the future.

Emerging evidences suggest that multiple signaling pathways activations and key transcription factors (TFs) are associated with the pathophysiology of DN, which could be influenced by epigenetically regulated mechanisms in chromatin (histones form a complex structure with DNA), including DNA methylation, posttranslational modifications (PTMs), and noncoding RNAs (ncRNA), which can modulate gene expression in the cell-type-specific pattern. Core histones are subject to diverse PTMs including histone lysine acetylation (HKAc), histone lysine methylation (HKme), phosphorylation, ubiquitination, and sumoylation. We have implicated the roles of HKme in the pathogenesis of DN [7], especially in the metabolic memory phenomenon pertinent to DN [2, 16, 17]. Global acetylation alterations have been seen in a lot of human diseases including cancer and nervous system diseases [18], whereas the roles of HAc in the pathogenesis of DN are rarely mentioned.

Recently, some studies showed that HAc level is linked to DN, HATs and HDACs also participate in the pathogenesis of DN, and the research regarding HAc and the covalent enzymes is not enough to yield a clear picture about DN so far. In this review, we describe some progress associated with the molecular mechanism underlying DN, with specific emphasis on HAc and acetylation on nonhistone proteins as important regulators of gene expression in renal cell under diabetic conditions; the regulators of HAc such as HATs as well as HDACs in the development and progression of DN; the inhibitors of HATs/HDACs in the DN pathogenesis and their therapeutic potentials for DN.

## 2. Histone and Nonhistone Acetylation in DN

Reversible acetylation of histones has been demonstrated for more than 50 years [19]. Dynamic balance of histone acetylation and deacetylation can regulate gene expression, chromosome assembly, mitosis, and PTMs [20], by altering the chromatin structure and the accessibility to TFs without affecting the sequence of DNA [21]. HAc is highly reversible and dynamic, which can be catalyzed by HATs or HDACs, respectively. HAc on H3 and H4 has been considered as marker of an “open” configuration of chromatin. HKAc at N-terminal tails can facilitate gene transcription through neutralizing the positive charge of histone residues and weakening the binding of histone to negatively charged DNA [22, 23]. HKAc, such as H3K9Ac, H3K14Ac, and H4KAc, is generally linked to permissive gene expression [24], while histone deacetylation is often associated with chromatin condensation and gene transcriptional repression [25, 26].

Several previous studies have shown that HKAc at the insulin gene promoter was specific to *β* cells and islet-derived precursor cells, which was highly correlated with the recruitment of p300 [27, 28].* In vitro* studies with HDAC inhibitors (HDACI) suggested that HKAc was essential in the development of pancreas [29]. These findings cannot fully demonstrate the underlying mechanism of DN; in this review, we will discuss the current opinions of HAc and nonhistone acetylation on inflammation, fibrosis, and oxidative stress in the development and progression of DN (Table 1).

Recent studies have demonstrated that dysregulated acetylation of core histone is associated with DN. Diabetic patients showed that levels of H3 acetylation at lysine 9 and 14 and H4 acetylation at lysine 5, 8, and 12 were increased at TNF-*α* and COX-2 inflammatory genes promoters in human blood monocytes [30]. Another study showed that oxidized lipids could increase H3K9/14Ac at MCP-1 and IL-6 gene promoters in a CREB/p300-dependent manner, along with the inflammatory genes expression [31]. Advanced DN in db/db mice underwent by uninephrectomy is specifically associated with increased acetylation of H3K9 and H3K23 [32]. A recent study revealed that acetylation of H3K9, H3K18, and H3K23 were significantly increased in the renal cortex of Akita mice, HG and NaB-induced H3K9 and H3K18 acetylation was elevated in the mesangial cells also, which were associated with inflammatory factors such as MCP-1, ICAM-1, VCAM-1, and iNOS expression linked to the development of DN [33]. HKAc mediated by HATs can increase transcriptional activity of proinflammatory NF-*κ*B under diabetic conditions [30]. Thioredoxin-interacting protein (TXNIP) has been demonstrated to play an important role in the pathogenesis of DN. HG-induced TXNIP expression was associated with the stimulation of activating H3K9Ac in MCs of diverse species, which could drive the expression of proinflammatory genes predisposing to DN [34].

TGF-*β*1 is established to be involved in the pathogenesis of DN, the underlying mechanism of which is still unclear. TGF-*β*1 treatment could increase acetylation of histone (H3K9, H3K14, and H3K27) as well as Ets-1 in mouse renal glomerular mesangial cells; furthermore, acetylation of Ets-1 and histone H3 was increased in glomeruli from diabetic db/db mice also, both of which can increase* miR-192* expression contributing to DN [35]. TGF-*β*1 can also mediate the effects of HG [2]. TGF-*β*1 treatment increased H3K9/14Ac at the PAI-1 and p21 promoters near Smad and SP1 binding sites in RMCs, acetylation of Smads was also increased [36, 37], and HG-treated RMCs exhibited increased levels of H3K9/14Ac that can be blocked by TGF-*β*1 antibodies, which played an important role in TGF-*β*1 and HG-induced deregulated gene expression associated with hypertrophy and fibrosis linked to DN [2]. HG stimulation can also increase H3K9/14Ac at the RAGE, PAI-1, and MCP-1 promoters, which can be further augmented by HG+Ang II (HG/A), suggesting the key roles of H3K9/14Ac in the key DN-related genes expression [38]. Excessive H3K9/14Ac levels were reported at the CTGF, PAI-1, and FN-1 promoters in diabetic kidneys, which were associated with p300/CBP activation [39]. Although there is a conflicting result in an animal study that the level of H3K9/14Ac was decreased in the STZ-induced type 1 diabetic rat kidney [40, 41], the majority of HAc is involved in the development and progression of DN.

For the past few years, the phenomenon “metabolic memory” has been implicated in the pathogenesis of diabetes and its complications such as DN. A study of patients from DCCT conventional treatment groups showed that there was association between HbA1c level and H3K9Ac; hyperacetylated promoters included more than 15 genes related to the NF-*κ*B pathway and could be enriched in genes associated with diabetic complications [42], which may be a possible epigenetic explanation along with HKme [16, 17, 43] for metabolic memory phenomenon in humans.

Endoplasmic reticulum stress (ERS) is an important mechanism responsible for the pathogenesis of DN. Histone H4 acetylation levels are increased at glucose-regulated protein (GRP78) promoters and decreased at C/EBP-homologous protein (CHOP) promoters, which are associated with renal cell apoptosis, proteinuria, and increases of Scr; these results provide initial experimental evidences for understanding the mechanism of DN [44].

Apart from HAc, nonhistone proteins acetylation can also take part in the pathogenesis of DN. Fork box O4 (Foxo4) transcription factor can be activated to promote podocyte apoptosis by AGEs through* Bcl2111* expression, at the same time, AGE-BSA can also increase Foxo4 acetylation; a recent study showed that alteration of Foxo4 acetylation and downregulation of Sirt1 expression in DM promote podocyte apoptosis; Foxo4 acetylation reduction could be a therapeutic potential for preventing diabetic podocyte loss [45]. Enhanced NF-*κ*B acetylation level was present in both diabetic rats and HG-treated RMC leading to DN in another study, which can be dampened by 3,5-diiodothyronine (T2) involved regulation of SIRT1 [46]; acetylation of NF-*κ*B p65 and STAT3 was increased in both mice and human diabetic kidneys and AGEs induced human podocytes, suggesting their critical roles in DN [47]. NF-*κ*B p65 acetylation was also increased by HG in RMCs, PNS could protect diabetic kidney through decreasing induction of inflammatory cytokines and TGF-*β*1 [48]. Smad 3 acetylation has been implicated in the pathogenesis of DN recently [49, 50], overexpression of transcription factor SREBP-1 induces glomerulosclerosis of DN; SREBP-1a K333 acetylation by CBP is required for Smad3 association and SREBP-1 transcriptional activity; both Smad3 and SREBP-1a activation regulates TGF-*β*1 transcriptional responses associated with DN, SREBP-1 inhibition could be a novel therapeutic strategy for DN [49]. Nephrin acetylation in diabetic podocytopathy has seldom been addressed before, a recent study showed that nephrin acetylation was reduced in STZ-induced diabetic mice kidney; increasing miR-29a may protect diabetic podocytopathy by modulating nephrin acetylation [51].

## 3. HATs and DN

There are two groups of HATs based on their cellular localizations. Type A HATs (nuclear) exist in nucleus, including (1) GNAT (GCN5) family such as GCN5, p/CAF, and ELP3, (2) MYST (HMOF/MYST1, HBO1/MYST2, MOZ/MYST3, MORF/MYST4, and TIP60) family, (3) p300/CBP, (4) basal TF family (TFIIIC and TAF1), and (5) NRCF family, SRC, and ACTR/NCOA3 [18], which can acetylate nucleosomal histones and other chromatin-associated proteins, while type B HATs are cytoplasmic and acetylate newly synthesized histones [52]. HKAc is generally mediated by HATs including p300, CBP, p/CAF, and TIP60, which is associated with gene activation via adding acetyl groups. In addition, HATs can also regulate gene expression through acetylation of nonhistone proteins such as Smads, p53, SP1, and NF-*κ*B.

Among the studies of HATs and their links with DN development,* in vitro* and* in vivo* studies showed that HATs CBP and p/CAF recruitment was increased under diabetic conditions, which led to upregulated HKAc at inflammatory genes promoters continent with the gene expression [30, 53]. It was implicated that p300 played important roles in oxidative stress-induced PARP and NF-*κ*B signaling in HG-treated endothelial cells and diabetic kidneys [53–55]; further study showed that HG upregulated p300, which increased HAc at promoters of key ECM protein FN, as well as vasoactive factors such as ET-1 and VEGF in endothelial cells [56]. Another study showed that TGF-*β*1 increased H3K9/14Ac by recruiting the HATs p300 and CBP; TGF-*β*1 treatment also increased association of p300 with Smad2/3 and SP1, cotransfection experiments showed that p300 and CBP, but not p/CAF, upregulated transcriptional activity of PAI-1 and p21 promoters and increased TGF-*β*1-induced gene expression. On the contrary, inhibition of CBP and p300 by overexpressing dominant-negative mutants could block TGF-*β*1-induced gene expression [36]. P/CAF was found sharply increased in the renal cortex of Akita mice, while GCN5 was significantly decreased in the HG group, suggesting that the inflammatory genes expressions were related to DN [33].* In vivo* and* in vitro* results of another report showed that p/CAF was closely related to H3K18Ac levels at inflammatory molecules ICAM-1 and MCP-1 promoters, which could be a potential therapeutic agent for inflammation-related renal diseases including DN [57]. All the data implied that HATs have critical roles in acetylating both histones and nonhistone proteins in the pathogenesis of DN; these results point to the necessity of further studies on the HATs activity in the development of DN, which may be therapeutic targets in the future.

## 4. HATs Inhibitors and DN

In preclinical trials, small-molecule HATs inhibitors have been shown to sensitize cancer cells to ionizing irradiation [58]. Curcumin, the p300/CBP inhibitor [59], extracted from rhizomes of turmeric* Curcuma longa* [60], which was supposed to be a new target molecule for treating CNS disorders and cancer [61, 62], was firstly reported to prevent the development of DN involved in the changes of PTMs of histone H3 including acetylation and phosphorylation and the changes in HSP-27 and p38 expression in diabetic rats [40]. Curcumin could also prevent HG-induced key ECM genes and vasoactive factors (eNOS and ET-1) expression levels associated with DN in endothelial cells [56]; it was able to reverse the upregulation of vasoactive factors, TGF-*β*1 and ECM protein FN in STZ-induced diabetic kidneys, which was associated with p300 and NF-*κ*B activity changes [63]. Curcumin was also found to reverse HG-induced cytokines (IL-6, TNF-*α*, and MCP-1) production in human monocytes via epigenetic changes involving NF-*κ*B [64], but dietary curcumin failed to decrease albuminuria either before or after diabetes induction [65]. Curcumin analogue, C66, has been demonstrated to significantly and persistently prevent renal injury and dysfunction in diabetic mice via downregulation of JNK activation and consequent suppression of diabetes-related increases in p300/CBP expression and histone acetylation (H3K9/14Ac) [39].

In a recent study, C646, a novel p300/CBP specific inhibitor, has been declared to specifically suppress the growth of CBP-deficient hematopoietic and lung cancer cells* in vivo* and* in vitro* [66]. In another* in vitro* study, histone H3Ac activated TGF-*β*1/Smad3 pathway during EMT of human peritoneal mesothelial cells; C646 could reverse the mesenchymal phenotype transition [67]. C646 was also reported reversing acetylation involved in HG-induced TXNIP expression leading to DN [34].

## 5. HDACs and DN

To date, 18 HDACs have been identified in humans and divided into 4 distinct classes based on their homology to yeast HDAC, in which Class I (HDAC1, 2, 3, and 8), Class II including IIa (HDAC4, 5, 7, and 9) and IIb (HDAC6 and 10), and Class IV (HDAC11) have structurally similar zinc-dependent active sites, whereas Class III, sirtuins (SIRTs1-7), are zinc-independent but require cofactor nicotinamide adenine dinucleotide (NAD) [52]. HDACs can remove acetyl groups from conserved lysine residues and nonhistone proteins and generally act as corepressors with some exceptions [2]. Evidence for mechanisms by which HDACs act in controlling DN is accumulating. Most research related to the epigenetics of DN has focused on HAc; different classes of HDACs are involved in distinct pathways that engaged in the pathogenesis of DN.

Overexpression of HDAC1 and HDAC5 blocked TGF-*β*1-induced gene expression, whereas inhibition of HDACs upregulated H3K9/14ac and gene expression, further supporting the key inhibitory roles of HDACs in TGF-*β*1-induced gene expression [36]. A recent study showed that HDAC1 was significantly decreased in the renal cortex of Akita mice, while the levels of HDAC2 in Akita and WT mice were unchanged, and HDAC1 was significantly decreased in HG-cultured HBZY-1 cell, which can upregulate diabetes-, HG-, and NaB-induced histone hyperacetylation leading to inflammatory factors elevation associated with DN [33].

Glomerular sclerosis is also a core characteristic of DN resulting from excessive ECM deposition in the glomerular mesangium and the loss of glomerular epithelial cells, followed by aberrant fibrosis in the glomerular structure. HDAC2 activity was markedly increased in the kidneys of type 1 and type 2 murine models and TGF-*β*1 treated NRK52-E cells, which played an important role in the development of DN [68]. Knockdown of HDAC2 in cell culture reduced ECM components accumulation, further implicating the role of HDAC2 in the fibrosis. Oxidative stress is also of the view to play an important role in regulating fibrosis in DN [69]; a potent oxidative stress inducer H_2_O_2_ can increase HDAC2 levels [68], which may be an underlying mechanism in the pathogenesis of DN.

HDAC4 is regarded as a contributor to podocyte injury in type 1 and type 2 diabetic models and diabetic patients and could suppress autophagy related with podocyte injury in DN by deacetylating STAT1, suggesting that HDAC4 is important to accelerate DN in epigenetic and nonepigenetic mechanisms [70, 71].

SIRTs have been shown to be involved in diverse cellular processes such as insulin secretion, cell cycle, and apoptosis [72]. Dysfunction of SIRT1 may contribute to abnormal cancer metabolism, cancer stemness, neurological disorders, obesity, and diabetes [72]. A previous study showed that decreased SIRT1 level in diabetic kidney and intermittent fasting (IF) prevents this decrease; SIRT1-dependent deacetylation is thought to mediate p53 expression and activation, which could play a renoprotective effect of IF in diabetes [73]. Another report showed that resveratrol could prevent decreased SIRT1 and increased p53 expression in diabetic kidney, which could be responsible for preventing apoptosis in type 1 diabetic kidney [74]. Resveratrol has also been demonstrated to reduce oxidative stress and maintain mitochondrial function related with SIRT1 activation in HG-treated MCs and* db/db* diabetic mice [75, 76]. SIRT1 in proximal tubules (PT) has been reported to attenuate diabetic albuminuria by suppressing the overexpression of tight junction protein Claudin-1 via hypermethylation of the Claudin-1 gene in podocytes [77, 78]. Another previous report showed that SIRT1 could inhibit TGF-*β*1-induced glomerular mesangial cell apoptosis via Smad7 deacetylation [79], and overexpression of SIRT1 attenuated ROS-induced apoptosis in mesangial cells through p53 deacetylation and provided a new therapeutic strategy for kidney glomerular diseases [80]; TSG has been proven to protect DN through inhibiting TGF-*β*1 expression partially mediated by SIRT1 activation [81]. Conditional SIRT1 deletion in podocytes of diabetic db/db mice developed more acetylation of NF-*κ*B p65 and STAT3, proteinuria, and kidney injury compared with db/db mice without SIRT1 deletion, suggesting the protective roles of SIRT1 in TFs acetylation on DN [47]. Dietary restriction was reported to ameliorate DN through regulation of the autophagy via restoration of SIRT1 in diabetic* fa/fa *rats [82]. The beneficial effects of SIRT1 on AGE-associated DN correlate with the activation of* Nrf2/ARE* antioxidative pathway [83, 84]. All the findings suggested the possibility of SIRT1 as the target of treatment in DN [85–87].

Taken together, these studies highlight important and different roles of HDACs in the pathways, and most of them are beneficial, suggesting HDACs will be the targets for the prevention of DN despite the fact that further studies are needed.

## 6. HDACIs and DN

The present HDACIs include both natural and synthetic compounds and are subdivided into 5 categories: short-chain fatty acids, cyclic peptides, benzamides, electrophilic ketones, and small-molecule hydroxamic-acid-derived compounds [52, 88]. HDACIs are regarded as potential anticancer agents and are promising for the treatment of a lot of diseases such as inflammation and neurological diseases [72]. Recently, HDACIs have been identified as a novel class of potential therapeutic agents for DN [89]. Here we list some progress of HDACIs applied in the treatment of DN regarding antifibrotic, anti-inflammatory, and antioxidative effects.

Nevertheless, most of the HDACIs are nonselective and target both nuclear histones and cytoplasmic nonhistone proteins [23]. It was found that millimolar concentrations of* n*-butyrate induce accumulations of acetylated histones in cells in the 1970s and inhibited deacetylation [72, 90, 91]. Sodium butyrate (NaB, a nonselective inhibitor of HDACs), a short-chain fatty acid, can upregulate HAc levels, promote tumor cell senescence and apoptosis, and inhibit tumor cell proliferation [20]. NaB was used as animal feed additive and played a major role in the treatment of neurodegenerative conditions.* In vivo*, it was reported that NaB could not only decrease blood glucose, creatinine, and urea but also ameliorate histological changes, fibrosis, apoptosis, and DNA damage in the kidneys of juvenile diabetic rats [92]. Further studies are needed to provide more evidences and theoretical basis in treating DN.

SAHA (suberoylanilide hydroxamic acid, vorinostat), a nonselective HDACI, designed and synthesized as a hybrid polar compound that can strongly induce erythroid differentiation [72, 93], is orally bioavailable and clinically applicable. SAHA can reduce albuminuria, glomerular hypertrophy, and glomerular type IV collagen deposition through an eNOS-dependent mechanism, without affecting blood pressure or blood glucose concentration [94]. Indeed, another study showed that SAHA attenuated early renal enlargement in STZ-induced diabetic rats, which is supposed to be mediated partly through downregulating EGFR [95]. These results indicated the key role of SAHA in attenuating fibrosis and oxidative damage in DN.

Trichostatin A (TSA), the natural product isolated from a* Streptomyces* strain, originally identified as an antifungal antibiotic, was discovered to have potent HDAC inhibition activity in 1990 [72]. TSA was reported to act as an agent in preventing DN in diabetic rats [32], by blocking TGF-*β*1-induced ECM accumulation [68] and EMT in diabetic kidneys [68] as well as in renal epithelial cells [96]; knockdown of HDAC2 had similar effect of TSA treatment mediated by ROS.

Valproic acid (VPA), a broad-spectrum HDACI, is a first-line drug used for the treatment of epilepsy and migraine. VPA treatment alleviated renal injury and fibrosis in STZ-induced diabetic kidney by preventing myofibroblast activation and fibrogenesis through HDAC4/5/7 inhibition in a dose-dependent manner [97], VPA has also been proven to ameliorate the podocyte and renal injuries by facilitating autophagy and inactivation of NF-*κ*B/iNOS pathway [98]. A recent study showed that VPA can attenuate renal injury in a rat model of DN, by upregulating the histone H4 acetylation levels at the promoter of GRP78 and downregulating the histone H4 acetylation at the promoter of CHOP [44].

To our knowledge, at the time of the present review, the molecular implications of HDACIs were identified in the treatment of DN, and the development of selective HDACIs in preventing DN may be part of the most prevalent areas in the drug discovery.

## 7. Conclusions and Perspectives

Recent research has concentrated on histone modifications to provide a reliable theoretical basis for clinical treatment. A comprehensive understanding of HAc mechanisms can give rise of novel therapeutic options for DN. Increasing* in vitro* and* in vivo* evidences implicated that reversible histone and nonhistone acetylation play important roles in the pathogenesis of DN, suggesting that HAc regulation could be promising therapeutic targets for DN. HATs and a small number of HDACs provide a central mechanism for regulating gene expression and cellular signaling events in DN (Table 2). Experimental evidences suggest that HATs/HDACs inhibitors and a large number of HDACs can delay the development and progression of DN (Tables 2 and 3). HATs inhibitor curcumin and its analogue C66 could protect renal injuries in diabetic patients and diabetic animal models; Apelin-13 and Esculetin treatment could be innovative therapeutic agents for DN via regulation of HAc also [33, 40, 41]. Continued research is needed to better understand the roles of HAc in the process of DN, the modifiers and the mechanism that regulate them, and address the curative potential of more selective HATs inhibitors and HDACI in treating DN.

## Figures and Tables

**Table 1:** Reported histone lysine and nonhistone acetylation in DN.

Ac proteins	Acetylation site	Target genes	Target renal loci	Effects in DN	References
Histone lysine	H3K9	MCP-1, ICAM-1, andVCAM-1; TXNIP	db/db mice kidney; Akita mice renal cortex, MCs	Advanced diabetic glomerulosclerosis; inflammation	[32–34]
	H3K9/14	TNF-*α*, COX-2; MCP-1, IL-6; PAI-1, p21; RAGE; CTGF, FN	Human blood monocytes; rat VSMCs; MMCs, db/db mice glomeruli; RMCs; STZ-induced mice	Inflammation; increased *miR-192* expression; hypertrophy, fibrosis	[30, 31, 35, 36, 38, 39]
	H3K18	MCP-1, ICAM-1, and VCAM-1	Akita mice renal cortex, MCs	Advanced diabetic glomerulosclerosis; inflammation	[33]
	H3K23		db/db mice kidney	Advanced diabetic glomerulosclerosis	[32, 33]
	H3K27		MMCs, db/db mice glomeruli	Increased *miR-192* expression	[35]
	H4	GRP78, CHOP	STZ-induced rat kidney	Cell apoptosis, proteinuria, and increase of Scr	[44]
	H4K5/8/12	TNF-*α*, COX-2	Human blood monocytes	Inflammation	[30]

Nonhistone proteins	Ets-1		MMCs, db/db mice glomeruli	Increased *miR-192* expression	[35]
	Foxo4	*Bcl2111*	Podocyte	Promoting apoptosis	[45]
	NF-*κ*B	TGF-*β*1, FN, and type IV collagen	RMCs, diabetic rats	UAE increase, matrix expansion, and ECM deposition	[46]
	NF-*κ*B p65	MCP-1, PAI-1, and TGF-*β*1	Mice and human diabetic kidneys, human podocytes; RMCs;	Kidney injury; inflammation;	[47, 48]
	STAT3		mice and human diabetic kidneys; human podocytes	Kidney injury	[47]
	Smad3	SREBP-1; type IV collagen	MMCs; iHMCs	Inducing glomerulosclerosis, increased albuminuria	[49, 50]
	Nephrin	WT-1, TGF-*β*1, and FN	STZ-induced FVB mice, podocyte	Ameliorate HG-induced podocyte dysfunction	[51]

**Table 2:** Reported HATs/HDACs in DN.

Enzyme category	Enzymes	Catalyzed site	Target renal loci	Effects in DN	References
HATs	CBP	H3K9/14, H4K5/8/12	Human monocytes; RMCs	Inflammation; increased TGF-*β*1-induced genes expression	[30, 36]
	GCN5		Akita mice renal cortex	Inflammation	[33]
	P300	H3K9/14	Endothelial cell, diabetic rats; RMCs	Inflammation, FN, vasoactive factors; increased TGF-*β*1-induced genes expression	[36, 54, 56]
	p/CAF	H3K9/14, H4K5/8/12; H3K18	Human monocytes; Akita mice renal cortex; db/db mice, human renal proximal tubule epithelial cell line	Inflammation	[30, 57]

HDACs	HDAC1	H3K9/14; H3K9, H3K18	RMCs; Akita mice, HBZY-1 cell	Blocking TGF-*β*1-induced gene expression; affecting inflammatory factors	[33, 36]
	HDAC2	H3/H4	Type1/2 murine models, NRK52-E cells	Promoting fibrosis	[68]
	HDAC4		Db/db mice, STZ-induced rats, diabetic patients	Contributing to podocyte injury	[70]
	HDAC5	H3K9/14	RMCs	Blocking TGF-*β*1-induced gene expression	[36]
	SIRT1	NF-*κ*B, STAT3	Renal tubular cells, podocyte; GMCs; db/db mice; diabetic *fa/fa *rats	Attenuating albuminuria; inhibiting cell apoptosis; attenuating kidney injury; regulating autophagy; reducing oxidative stress	[47, 75–80, 82–84]

**Table 3:** Effect of inhibitors of HATs/HDACs in DN.

Inhibitors category	Name	Target genes	Target renal loci	Effects in DN	References
HATs inhibitors	Curcumin	ECM genes, vasoactive factors; inflammatory genes;	STZ-induced rats; endothelial cell; human monocytes	Reversing ECM proteins and vasoactive factors upregulation; reverse HG-induced cytokines	[40, 56, 63, 64]
	C66	CTGF, PAI-1, and FN-1	STZ-induced mice	Preventing renal fibrosis and dysfunction	[39]
	C646	TXNIP	Diabetic Sur1-E1506K(+/+) mice	Reversing acetylation leading to DN	[34]

HDACs inhibitors	NaB		Juvenile diabetic rats	Decreasing blood glucose, creatinine, and urea; ameliorating histological changes, fibrosis, and apoptosis	[92]
	SAHA	type IV collagen	STZ-induced mice, HUVECs; STZ-induced rats, NRK	Decreasing albuminuria, glomerular hypertrophy	[94, 95]
	TSA		STZ-induced rats, NRK52-E	Blocking TGF-*β*1 induced ECM accumulation and EMT	[68, 96]
	VPA	TGF-*β*1, CTGF, FN, collagen I, COX-2, and ICAM-1	STZ-induced diabetic rats	Alleviating renal injury and fibrosis; ameliorating podocyte and renal injury	[97, 98]

## References

[1] Tervaert T. W. C., Mooyaart A. L., Amann K. (2010). Pathologic classification of diabetic nephropathy. *Journal of the American Society of Nephrology*.

[2] Reddy M. A., Tak Park J., Natarajan R. (2013). Epigenetic modifications in the pathogenesis of diabetic nephropathy. *Seminars in Nephrology*.

[3] Matsushita K., van der Velde M., Astor B. C. (2010). Association of estimated glomerular filtration rate and albuminuria with all-cause and cardiovascular mortality in general population cohorts: a collaborative meta-analysis. *The Lancet*.

[4] Forbes J. M., Cooper M. E. (2013). Mechanisms of diabetic complications. *Physiological Reviews*.

[5] Yacoub R., Campbell K. N. (2015). Inhibition of RAS in diabetic nephropathy. *International Journal of Nephrology and Renovascular Disease*.

[6] Kolset S. O., Reinholt F. P., Jenssen T. (2012). Diabetic nephropathy and extracellular matrix. *The Journal of Histochemistry and Cytochemistry*.

[7] Sun G.-D., Cui W.-P., Guo Q.-Y., Miao L.-N. (2014). Histone lysine methylation in diabetic nephropathy. *Journal of Diabetes Research*.

[8] Chokhandre M. K., Mahmoud M. I., Hakami T., Jafer M., Inamdar A. S. (2015). Vitamin D & its analogues in type 2 diabetic nephropathy: a systematic review. *Journal of Diabetes and Metabolic Disorders*.

[9] Wada J., Makino H. (2013). Inflammation and the pathogenesis of diabetic nephropathy. *Clinical Science*.

[10] Mezzano S., Aros C., Droguett A. (2004). NF-*κ*B activation and overexpression of regulated genes in human diabetic nephropathy. *Nephrology Dialysis Transplantation*.

[11] Hasegawa G., Nakano K., Sawada M. (1991). Possible role of tumor necrosis factor and interleukin-1 in the development of diabetic nephropathy. *Kidney International*.

[12] Lin M., Yiu W. H., Wu H. J. (2012). Toll-like receptor 4 promotes tubular inflammation in diabetic nephropathy. *Journal of the American Society of Nephrology*.

[13] Singh R., Alavi N., Singh A. K., Leehey D. J. (1999). Role of angiotensin II in glucose-induced inhibition of mesangial matrix degradation. *Diabetes*.

[14] Kato M., Natarajan R. (2014). Diabetic nephropathy—emerging epigenetic mechanisms. *Nature Reviews Nephrology*.

[15] Kato M., Natarajan R. (2015). MicroRNAs in diabetic nephropathy: functions, biomarkers, and therapeutic targets. *Annals of the New York Academy of Sciences*.

[16] Tonna S., El-Osta A., Cooper M. E., Tikellis C. (2010). Metabolic memory and diabetic nephropathy: potential role for epigenetic mechanisms. *Nature Reviews: Nephrology*.

[17] Brasacchio D., Okabe J., Tikellis C. (2009). Hyperglycemia induces a dynamic cooperativity of histone methylase and demethylase enzymes associated with gene-activating epigenetic marks that coexist on the lysine tail. *Diabetes*.

[18] Wang Y., Miao X., Liu Y. (2014). Dysregulation of histone acetyltransferases and deacetylases in cardiovascular diseases. *Oxidative Medicine and Cellular Longevity*.

[19] Waterborg J. H. (2002). Dynamics of histone acetylation in vivo. A function for acetylation turnover?. *Biochemistry and Cell Biology*.

[20] Zhang H., Dai X., Qi Y., He Y., Du W., Pang J.-J. (2015). Histone deacetylases inhibitors in the treatment of retinal degenerative diseases: overview and perspectives. *Journal of Ophthalmology*.

[21] Egger G., Liang G., Aparicio A., Jones P. A. (2004). Epigenetics in human disease and prospects for epigenetic therapy. *Nature*.

[22] Kouzarides T. (2007). Chromatin modifications and their function. *Cell*.

[23] Lu X., Wang L., Yu C., Yu D., Yu G. (2015). Histone acetylation modifiers in the pathogenesis of Alzheimer’s disease. *Frontiers in Cellular Neuroscience*.

[24] Reddy M. A., Natarajan R. (2011). Epigenetics in diabetic kidney disease. *Journal of the American Society of Nephrology*.

[25] Campos E. I., Reinberg D. (2009). Histones: annotating chromatin. *Annual Review of Genetics*.

[26] Mathiyalagan P., Keating S. T., Du X.-J., El-Osta A. (2014). Chromatin modifications remodel cardiac gene expression. *Cardiovascular Research*.

[27] Chakrabarti S. K., Francis J., Ziesmann S. M., Garmey J. C., Mirmira R. G. (2003). Covalent histone modifications underlie the developmental regulation of insulin gene transcription in pancreatic *β* cells. *The Journal of Biological Chemistry*.

[28] Mutskov V., Raaka B. M., Felsenfeld G., Gershengorn M. C. (2007). The human insulin gene displays transcriptionally active epigenetic marks in islet-derived mesenchymal precursor cells in the absence of insulin expression. *STEM CELLS*.

[29] Haumaitre C., Lenoir O., Scharfmann R. (2008). Histone deacetylase inhibitors modify pancreatic cell fate determination and amplify endocrine progenitors. *Molecular and Cellular Biology*.

[30] Miao F., Gonzalo I. G., Lanting L., Natarajan R. (2004). In vivo chromatin remodeling events leading to inflammatory gene transcription under diabetic conditions. *The Journal of Biological Chemistry*.

[31] Reddy M. A., Sahar S., Villeneuve L. M., Lanting L., Natarajan R. (2009). Role of Src tyrosine kinase in the atherogenic effects of the 12/15-lipoxygenase pathway in vascular smooth muscle cells. *Arteriosclerosis, Thrombosis, and Vascular Biology*.

[32] Sayyed S. G., Gaikwad A. B., Lichtnekert J. (2010). Progressive glomerulosclerosis in type 2 diabetes is associated with renal histone H3K9 and H3K23 acetylation, H3K4 dimethylation and phosphorylation at serine 10. *Nephrology Dialysis Transplantation*.

[33] Chen H., Li J., Jiao L. (2014). Apelin inhibits the development of diabetic nephropathy by regulating histone acetylation in Akita mouse. *Journal of Physiology*.

[34] De Marinis Y., Cai M., Bompada P. (2016). Epigenetic regulation of the thioredoxin-interacting protein (TXNIP) gene by hyperglycemia in kidney. *Kidney International*.

[35] Kato M., Dang V., Wang M. (2013). TGF-*β* induces acetylation of chromatin and of Ets-1 to alleviate repression of miR-192 in diabetic nephropathy. *Science Signaling*.

[36] Yuan H., Reddy M. A., Sun G. (2013). Involvement of p300/CBP and epigenetic histone acetylation in TGF-*β*1-mediated gene transcription in mesangial cells. *American Journal of Physiology—Renal Physiology*.

[37] Das F., Ghosh-Choudhury N., Venkatesan B., Li X., Mahimainathan L., Choudhury G. G. (2008). Akt kinase targets association of CBP with SMAD 3 to regulate TGF*β*-induced expression of plasminogen activator inhibitor-1. *Journal of Cellular Physiology*.

[38] Reddy M. A., Sumanth P., Lanting L. (2014). Losartan reverses permissive epigenetic changes in renal glomeruli of diabetic db/db mice. *Kidney International*.

[39] Wang Y., Wang Y., Luo M. (2015). Novel curcumin analog C66 prevents diabetic nephropathy via JNK pathway with the involvement of p300/CBP-mediated histone acetylation. *Biochimica et Biophysica Acta—Molecular Basis of Disease*.

[40] Tikoo K., Meena R. L., Kabra D. G., Gaikwad A. B. (2008). Change in post-translational modifications of histone H3, heat-shock protein-27 and MAP kinase p38 expression by curcumin in streptozotocin-induced type I diabetic nephropathy. *British Journal of Pharmacology*.

[41] Surse V. M., Gupta J., Tikoo K. (2011). Esculetin induced changes in Mmp13 and Bmp6 gene expression and histone H3 modifications attenuate development of glomerulosclerosis in diabetic rats. *Journal of Molecular Endocrinology*.

[42] Miao F., Chen Z., Genuth S. (2014). Evaluating the role of epigenetic histone modifications in the metabolic memory of type 1 diabetes. *Diabetes*.

[43] El-Osta A., Brasacchio D., Yao D. (2008). Transient high glucose causes persistent epigenetic changes and altered gene expression during subsequent normoglycemia. *The Journal of Experimental Medicine*.

[44] Sun X. Y., Qin H. J., Zhang Z. (2016). Valproate attenuates diabetic nephropathy through inhibition of endoplasmic reticulum stressinduced apoptosis. *Molecular Medicine Reports*.

[45] Chuang P. Y., Dai Y., Liu R. (2011). Alteration of forkhead box o (foxo4) acetylation mediates apoptosis of podocytes in diabetes mellitus. *PLoS ONE*.

[46] Shang G., Gao P., Zhao Z. (2013). 3,5-Diiodo-l-thyronine ameliorates diabetic nephropathy in streptozotocin-induced diabetic rats. *Biochimica et Biophysica Acta—Molecular Basis of Disease*.

[47] Liu R., Zhong Y., Li X. (2014). Role of transcription factor acetylation in diabetic kidney disease. *Diabetes*.

[48] Du Y.-G., Wang L.-P., Qian J.-W., Zhang K.-N., Chai K.-F. (2015). *Panax notoginseng* saponins protect kidney from diabetes by up-regulating silent information regulator 1 and activating antioxidant proteins in rats. *Chinese Journal of Integrative Medicine*.

[49] Chen G., Wang T., Uttarwar L. (2014). SREBP-1 is a novel mediator of TGF*β*1 signaling in mesangial cells. *Journal of Molecular Cell Biology*.

[50] Papadimitriou A., Silva K. C., Peixoto E. B. M. I., Borges C. M., de Faria J. M. L., de Faria J. B. L. (2015). Theobromine increases NAD+/Sirt-1 activity and protects the kidney under diabetic conditions. *American Journal of Physiology—Renal Physiology*.

[51] Lin C.-L., Lee P.-H., Hsu Y.-C. (2014). MicroRNA-29a promotion of nephrin acetylation ameliorates hyperglycemia-induced podocyte dysfunction. *Journal of the American Society of Nephrology*.

[52] Keppler B. R., Archer T. K. (2008). Chromatin-modifying enzymes as therapeutic targets—part 1. *Expert Opinion on Therapeutic Targets*.

[53] Villeneuve L. M., Natarajan R. (2010). The role of epigenetics in the pathology of diabetic complications. *American Journal of Physiology—Renal Physiology*.

[54] Kaur H., Chen S., Xin X., Chiu J., Khan Z. A., Chakrabarti S. (2006). Diabetes-induced extracellular matrix protein expression is mediated by transcription coactivator p300. *Diabetes*.

[55] Xu B., Chiu J., Feng B., Chen S., Chakrabarti S. (2008). PARP activation and the alteration of vasoactive factors and extracellular matrix protein in retina and kidney in diabetes. *Diabetes/Metabolism Research and Reviews*.

[56] Chen S., Feng B., George B., Chakrabarti R., Chen M., Chakrabarti S. (2010). Transcriptional coactivator p300 regulates glucose-induced gene expression in endothelial cells. *American Journal of Physiology—Endocrinology and Metabolism*.

[57] Huang J., Wan D., Li J., Chen H., Huang K., Zheng L. (2015). Histone acetyltransferase PCAF regulates inflammatory molecules in the development of renal injury. *Epigenetics*.

[58] Oike T., Ogiwara H., Amornwichet N., Nakano T., Kohno T. (2014). Chromatin-regulating proteins as targets for cancer therapy. *Journal of Radiation Research*.

[59] Balasubramanyam K., Varier R. A., Altaf M. (2004). Curcumin, a novel p300/CREB-binding protein-specific inhibitor of acetyltransferase, represses the acetylation of histone/nonhistone proteins and histone acetyltransferase-dependent chromatin transcription. *The Journal of Biological Chemistry*.

[60] Zammataro M., Sortino M. A., Parenti C., Gereau R. W., Chiechio S. (2014). HDAC and HAT inhibitors differently affect analgesia mediated by group II metabotropic glutamate receptors. *Molecular Pain*.

[61] Kang S.-K., Cha S.-H., Jeon H.-G. (2006). Curcumin-induced histone hypoacetylation enhances caspase-3-dependent glioma cell death and neurogenesis of neural progenitor cells. *Stem Cells and Development*.

[62] Manzo F., Tambaro F. P., Mai A., Altucci L. (2009). Histone acetyltransferase inhibitors and preclinical studies. *Expert Opinion on Therapeutic Patents*.

[63] Chiu J., Khan Z. A., Farhangkhoee H., Chakrabarti S. (2009). Curcumin prevents diabetes-associated abnormalities in the kidneys by inhibiting p300 and nuclear factor-*κ*B. *Nutrition*.

[64] Yun J.-M., Jialal I., Devaraj S. (2011). Epigenetic regulation of high glucose-induced proinflammatory cytokine production in monocytes by curcumin. *The Journal of Nutritional Biochemistry*.

[65] Ma J., Phillips L., Wang Y. (2010). Curcumin activates the p38MPAK-HSP25 pathway in vitro but fails to attenuate diabetic nephropathy in DBA2J mice despite urinary clearance documented by HPLC. *BMC Complementary and Alternative Medicine*.

[66] Ogiwara H., Sasaki M., Mitachi T. (2016). Targeting p300 addiction in CBP-deficient cancers causes synthetic lethality by apoptotic cell death due to abrogation of MYC expression. *Cancer Discovery*.

[67] Yang Y., Liu K., Liang Y., Chen Y., Chen Y., Gong Y. (2015). Histone acetyltransferase inhibitor C646 reverses epithelial to mesenchymal transition of human peritoneal mesothelial cells via blocking TGF-*β*1/Smad3 signaling pathway in vitro. *International Journal of Clinical and Experimental Pathology*.

[68] Noh H., Oh E. Y., Seo J. Y. (2009). Histone deacetylase-2 is a key regulator of diabetes- and transforming growth factor-beta1-induced renal injury. *American Journal of Physiology—Renal Physiology*.

[69] Rhyu D. Y., Yang Y., Ha H. (2005). Role of reactive oxygen species in TGF-*β*1-induced mitogen-activated protein kinase activation and epithelial-mesenchymal transition in renal tubular epithelial cells. *Journal of the American Society of Nephrology*.

[70] Wang X., Liu J., Zhen J. (2014). Histone deacetylase 4 selectively contributes to podocyte injury in diabetic nephropathy. *Kidney International*.

[71] Wei Q., Dong Z. (2014). HDAC4 blocks autophagy to trigger podocyte injury: non-epigenetic action in diabetic nephropathy. *Kidney International*.

[72] Seto E., Yoshida M. (2014). Erasers of histone acetylation: the histone deacetylase enzymes. *Cold Spring Harbor Perspectives in Biology*.

[73] Tikoo K., Tripathi D. N., Kabra D. G., Sharma V., Gaikwad A. B. (2007). Intermittent fasting prevents the progression of type I diabetic nephropathy in rats and changes the expression of Sir2 and p53. *FEBS Letters*.

[74] Tikoo K., Singh K., Kabra D., Sharma V., Gaikwad A. (2008). Change in histone H3 phosphorylation, MAP kinase p38, SIR 2 and p53 expression by resveratrol in preventing streptozotocin induced type I diabetic nephropathy. *Free Radical Research*.

[75] Xu Y., Nie L., Yin Y.-G. (2012). Resveratrol protects against hyperglycemia-induced oxidative damage to mitochondria by activating SIRT1 in rat mesangial cells. *Toxicology and Applied Pharmacology*.

[76] Kim M. Y., Lim J. H., Youn H. H. (2013). Resveratrol prevents renal lipotoxicity and inhibits mesangial cell glucotoxicity in a manner dependent on the AMPK-SIRT1-PGC1*α* axis in db/db mice. *Diabetologia*.

[77] Bible E. (2013). Diabetic nephropathy: sirt1 attenuates diabetic albuminuria. *Nature Reviews Nephrology*.

[78] Hasegawa K., Wakino S., Simic P. (2013). Renal tubular sirt1 attenuates diabetic albuminuria by epigenetically suppressing Claudin-1 overexpression in podocytes. *Nature Medicine*.

[79] Kume S., Haneda M., Kanasaki K. (2007). SIRT1 inhibits transforming growth factor *β*-induced apoptosis in glomerular mesangial cells via Smad7 deacetylation. *Journal of Biological Chemistry*.

[80] Kume S., Haneda M., Kanasaki K. (2006). Silent information regulator 2 (SIRT1) attenuates oxidative stress-induced mesangial cell apoptosis via p53 deacetylation. *Free Radical Biology and Medicine*.

[81] Li C., Cai F., Yang Y. (2010). Tetrahydroxystilbene glucoside ameliorates diabetic nephropathy in rats: involvement of SIRT1 and TGF-*β*1 pathway. *European Journal of Pharmacology*.

[82] Kitada M., Takeda A., Nagai T., Ito H., Kanasaki K., Koya D. (2011). Dietary restriction ameliorates diabetic nephropathy through anti-inflammatory effects and regulation of the autophagy via restoration of sirt1 in diabetic wistar fatty (*fa/fa*) rats: a model of type 2 diabetes. *Experimental Diabetes Research*.

[83] Huang K., Huang J., Xie X. (2013). Sirt1 resists advanced glycation end products-induced expressions of fibronectin and TGF-*β*1 by activating the Nrf2/ARE pathway in glomerular mesangial cells. *Free Radical Biology and Medicine*.

[84] Huang K., Chen C., Hao J. (2015). Polydatin promotes Nrf2-ARE anti-oxidative pathway through activating Sirt1 to resist AGEs-induced upregulation of fibronetin and transforming growth factor-*β*1 in rat glomerular messangial cells. *Molecular and Cellular Endocrinology*.

[85] Kume S., Kitada M., Kanasaki K., Maegawa H., Koya D. (2013). Anti-aging molecule, Sirt1: a novel therapeutic target for diabetic nephropathy. *Archives of Pharmacal Research*.

[86] Polak-Jonkisz D., Laszki-Szczachor K., Rehan L., Pilecki W., Filipowski H., Sobieszczańska M. (2013). Nephroprotective action of sirtuin 1 (SIRT1). *Journal of Physiology and Biochemistry*.

[87] Dong Y.-J., Liu N., Xiao Z. (2014). Renal protective effect of sirtuin 1. *Journal of Diabetes Research*.

[88] Wagner F. F., Wesmall yi U. M., Lewis M. C., Holson E. B. (2013). Small molecule inhibitors of zinc-dependent histone deacetylases. *Neurotherapeutics*.

[89] Lee H. B., Noh H., Seo J. Y., Yu M. R., Ha H. (2007). Histone deacetylase inhibitors: a novel class of therapeutic agents in diabetic nephropathy. *Kidney International Supplement*.

[90] Riggs M. G., Whittaker R. G., Neumann J. R., Ingram V. M. (1977). n-Butyrate causes histone modification in HeLa and friend erythroleukaemia cells. *Nature*.

[91] Candido E. P. M., Reeves R., Davie J. R. (1978). Sodium butyrate inhibits histone deacetylation in cultured cells. *Cell*.

[92] Khan S., Jena G. (2014). Sodium butyrate, a HDAC inhibitor ameliorates eNOS, iNOS and TGF-*β*1-induced fibrogenesis, apoptosis and DNA damage in the kidney of juvenile diabetic rats. *Food and Chemical Toxicology*.

[93] Richon V. M., Emiliani S., Verdin E. (1998). A class of hybrid polar inducers of transformed cell differentiation inhibits histone deacetylases. *Proceedings of the National Academy of Sciences of the United States of America*.

[94] Advani A., Huang Q., Thai K. (2011). Long-term administration of the histone deacetylase inhibitor vorinostat attenuates renal injury in experimental diabetes through an endothelial nitric oxide synthase-dependent mechanism. *The American Journal of Pathology*.

[95] Gilbert R. E., Huang Q., Thai K. (2011). Histone deacetylase inhibition attenuates diabetes-associated kidney growth: potential role for epigenetic modification of the epidermal growth factor receptor. *Kidney International*.

[96] Yoshikawa M., Hishikawa K., Marumo T., Fujita T. (2007). Inhibition of histone deacetylase activity suppresses epithelial-to- mesenchymal transition induced by TGF-*β*1 in human renal epithelial cells. *Journal of the American Society of Nephrology*.

[97] Khan S., Jena G., Tikoo K. (2015). Sodium valproate ameliorates diabetes-induced fibrosis and renal damage by the inhibition of histone deacetylases in diabetic rat. *Experimental and Molecular Pathology*.

[98] Khan S., Jena G., Tikoo K., Kumar V. (2015). Valproate attenuates the proteinuria, podocyte and renal injury by facilitating autophagy and inactivation of NF-*κ*B/iNOS signaling in diabetic rat. *Biochimie*.

